# The keratin–desmosome scaffold: pivotal role of desmosomes for keratin network morphogenesis

**DOI:** 10.1007/s00018-019-03198-y

**Published:** 2019-06-26

**Authors:** Marcin Moch, Nicole Schwarz, Reinhard Windoffer, Rudolf E. Leube

**Affiliations:** Institute of Molecular and Cellular Anatomy, RWTH Aachen University, Wendlingweg 2, 52074 Aachen, Germany

**Keywords:** Cytoskeleton, Intermediate filament, Desmosome, Keratin, Assembly, Cell-adhesion, Blastocyst, Keratinocyte, Live cell imaging

## Abstract

Desmosome-anchored keratin intermediate filaments (KFs) are essential for epithelial coherence. Yet, desmosomal KF attachment and network organization are still unexplored in vivo. We, therefore, monitored KF network morphogenesis in fluorescent keratin 8 knock-in murine embryos revealing keratin enrichment at newly formed desmosomes followed by KF formation, KF elongation and KF fusion. To examine details of this process and its coupling to desmosome formation, we studied fluorescent keratin and desmosomal protein reporter dynamics in the periphery of expanding HaCaT keratinocyte colonies. Less than 3 min after the start of desmosomal proteins clustering non-filamentous keratin enriched at these sites followed by KF formation and elongation. Subsequently, desmosome-anchored KFs merged into stable bundles generating a rim-and-spokes system consisting of subcortical KFs connecting desmosomes to each other and radial KFs connecting desmosomes to the cytoplasmic KF network. We conclude that desmosomes are organizing centers for the KF cytoskeleton with a hitherto unknown nucleation capacity.

**Electronic supplementary material:**

The online version of this article (10.1007/s00018-019-03198-y) contains supplementary material, which is available to authorized users.

## Introduction

The desmosome-anchored keratin intermediate filament (KF) cytoskeleton is subject to and protects against mechanical stress. On a structural level the keratin–desmosome scaffold is assembled from type I and type II keratins that directly bind to the desmosomal plaque protein desmoplakin [1–3]. This connection is regulated by posttranslational modification [4] and is further stabilized by contributions of the desmosomal plaque proteins plakophilin (Pkp) 1–3 [5–8]. Pkps1–3 and the structurally related universal adherens junction protein plakoglobin (Pg), in turn, bind to desmosomal cadherins of the desmocollin (Dsc) and desmoglein (Dsg) type, which mediate the transcellular contact to adjacent cells bearing identical desmosomal half structures with associated KF bundles (cf. [9]).

Despite this well-established connectivity of the keratin–desmosome scaffold, surprisingly little is known about its formation. Electron microscopy of developing embryos showed that desmosomes and KFs appear together in the trophectoderm of developing blastocysts [10]. These observations were recently extended to living embryos by time-lapse fluorescence microscopy of fluorescently tagged keratin 8, which enriched at distinct desmoplakin-positive plasma membrane sites [11]. The literature on simultaneous monitoring of keratin network and desmosome organization is rather scarce and does not explain how keratins are recruited to desmosomes (e.g., [12–14]). A possible reason for this apparent lack of research activities may be the fact that desmosomes and keratin networks can form independently of each other in cell culture and in tissues (e.g., [15–17]). Yet, deletion of keratins leads to reduced desmosomal size, increased desmosomal motility and reduced adhesion [15]. Conversely, disruption of desmosomes perturbs KF network organization [17, 18].

Examination of the processes underlying desmosome formation has been subject of research for many decades (cf. [9]). The emerging scenario reveals a situation, whereby the different molecular components are transported separately to the cell periphery for locally restricted and sequential assembly into maturing desmosomal adhesion sites (reviewed by [19]). Thus, Dsg2 and Dsc2 are transported by different microtubule-associated motors to the plasma membrane [19, 20]. The subsequent order of clustering and recruitment of desmosomal cadherins from cholesterol-rich raft domains is not fully understood [21–23]. But it has been proposed that E-cadherin together with Dsg2 initiates the recruitment of Pg–Pkp (isoforms 2 and 3) complexes to newly forming cell–cell contacts [24, 25]. It is also known that cytoplasmic Pkp2–desmoplakin complexes are delivered to the plasma membrane in an actin-dependent transport [26]. While details of the individual steps of this model still require additional experimental confirmation, even less is known, how and when the assembling desmosomes attach to the cytoplasmic intermediate cytoskeleton. Furthermore, the inception of KF formation is only in part understood. De novo assembly has been described to occur in the periphery of desmosome-free single cells in culture. The nucleating particles subsequently grow into small filaments that are transported toward the cell interior, where they integrate into the pre-existing network as part of a multistep turnover cycle. This turnover mechanism, referred to as the “keratin cycle”, is complemented by direct exchange of soluble keratin subunits throughout the keratin network [27]. Details of keratin network dynamics in desmosome-bearing cell assemblies, however, have not been examined in detail [28, 29].

The aim of the current study was to determine, where and how desmosome formation and KF recruitment occur in coupled cells and how the association of both affects their maturation and stability. To address these questions, we investigated the assembly of the first keratin network in keratin 8-EYFP mouse embryos and studied the observed phenomena in more detail in immortalized HaCaT keratinocytes.

## Materials and methods

### Mouse embryos

Keratin 8-EYFP knock-in mice have been previously generated [11]. Isolation of preimplantation embryos and imaging procedures has been described in detail [30]. In short, embryos were flushed out of the oviducts of pregnant mice on day E2.5 and kept in culture overnight in M16 medium (Sigma-Aldrich) at 37 °C. Only embryos that developed into morulae or blastocysts were used. Mice were housed in the animal facility of the RWTH Aachen University Hospital. All experiments were performed in accordance with guidelines for the care and use of laboratory animals which follow the latest version of the German animal protection law.

### Cell culture

Immortalized human HaCaT keratinocytes were kindly provided by Dr. Petra Boukamp [31] and were used to generate a HK5-EYFP expressing single cell clone B10 [32]. The cells were grown at 37 °C in a 5% CO_2_ humidified atmosphere and DMEM containing l-alanyl-glutamine (Sigma-Aldrich) and 9% (v/v) fetal bovine serum SeraPlus (PAN Biotech). For passaging, cells were washed and incubated for 15 min in PBS without Ca^2+^/Mg^2+^ (Sigma-Aldrich) and thereafter trypsinized for ≈ 5 min in a solution of PBS without Ca^2+^/Mg^2+^ (Biochrom) containing 0.25% (w/v) trypsin (Biochrom) supplemented with 0.02% (w/v) EDTA (Sigma-Aldrich). Cells were passaged once per week 1 day after reaching confluence and were seeded at a concentration of 40,000–60,000 cells/cm^2^ for DNA transfection. Cells were transfected on day 2 after seeding with 5 µg of plasmid DNA and 1.5 µl Xfect (Takara) in a total volume of 100 µl Xfect reaction buffer per 2 ml of cell culture medium in 35-mm diameter dishes.

### DNA cloning

To prepare a Dsc2-mCerulean-encoding plasmid, nucleotides coding for mCherry were first excised by *Bam*HI/*Not*I from plasmid pRSET_B_ (gift from Roger Tsien, [33]) and were inserted into plasmid pEYFP-N1 (Clontech). Resulting plasmid pmCherry-N1 was then subjected to *Bam*HI/*Xho*I restriction for subsequent insertion of the human DSC2 cDNA (GenBank id: BC063291.1) from plasmid dsc2a.EGFP-N1 [13] yielding plasmid DSC2-mCherry. Next, the mCerulean-encoding sequence was PCR amplified from Addgene plasmid #15214 (gift from Dave Piston, [34]) using primers 5′-TGGCCAGGATCCGATGGTGAGCAAGGGCGAGGAG-3′ and 5′-CGGACTTGTACAGCTCGTCCATG-3′. The PCR-product was digested with *Bam*HI/*Bsr*GI and inserted into plasmid DSC2-mCherry replacing the mCherry-sequence and thereby generating plasmid DSC2-mCerulean.

The human keratin 14 cDNA (gift from Werner Franke; GenBank id: BC042437.1) was PCR amplified using primers 5′-AAAAAGCTTATGACTACCTGCAGCCGCCAG-3′ and 5′-AAAGGATCCGGGTTCTTGGTGCGAAGGACCTG-3′, restricted with *Bam*HI/*Hin*dIII and inserted into the *Bam*HI/*Hin*dIII sites of pEYFP-N1 (Clontech) producing plasmid C-HK14-EYFP. Next, the keratin 14 cDNA was excised with *Bam*HI/*Not*I and used to replace the DSC2 cDNA in DSC2-mCerulean producing plasmid K14-mCerulean.

The human DSG2 cDNA flanked by *Xho*I/*Bsr*GI-restriction sites was a gift from Stephan Schäfer (DKFZ, Heidelberg). The DSG2-sequence corresponds to GenBank id: BC099655.3 but lacks the codons for the carboxyterminal amino acids QHSYS. The DSG2-sequence was excised by *Xho*I/*Bsr*GI restriction and ligated into the compatible *Xho*I/*Asp*718 I sites of pEGFP-N1 (Clontech) to generate DSG2-EGFP. From this plasmid the DSG2-sequence was excised by *Xho*I/*Sac*II restriction and was subsequently cloned into pmCherry-N1 producing plasmid DSG2-mCherry.

Plasmid p928 coding for human DSPI fused to EGFP was a generous gift from Kathy Green [26]. To produce an mApple version plasmid K13dT-mApple was first prepared. To this end, the EGFP-encoding region of HK13-EGFP [35] was removed by *Bam*HI/*Bsr*GI restriction and was replaced by a *Bam*HI/*Bsr*GI-restricted mApple-sequence that had been PCR-amplified with primers 5′-CGGGATCCATCGCCACCATGGTGAGCAAG-3′ and 5′-TCCGGACTTGTACAGCTCGTCCAT-3′ from an mApple-actin plasmid kindly provided by James Nelson. Next, pmApple-N1 was generated by removal of the mCherry-sequence from plasmid pmCherry-N1 by *Not*I/*Bam*HI restriction and replacement with the *Not*I/*Bam*HI mApple-encoding sequence from K13dT-mApple. The DSPI-encoding sequence was then excised from plasmid p928 by *Nhe*I/*Age*I restriction and was ligated into the compatible *Nhe*I/*Xma*I sites of pmApple-N1. The DSPI-sequence in resulting plasmid DSPI-mApple corresponds to GenBank id: NM_004415.3. All inserted sequences were verified by sequencing (Eurofins MWG).

### Immunocytochemistry and cell fixation

HaCaT cells were grown on 18-mm diameter high-precision glass cover slips with a thickness of 170 µm (Paul Marienfeld) at a concentration of ≈ 100,000 cells/cm^2^ in six-well dishes (CytoOne). Fixation for immunocytochemistry was performed by incubation in methanol for 3 min at − 20 °C followed by a 30-s immersion in acetone at − 20 °C. Fixed cells were washed in PBS (Biochrom) for 5 min and incubated with primary antibodies for 1 h, washed with PBS for 15 min, and incubated in PBS with secondary antibodies and 0.2 µg/ml 4′,6-diamidino-2-phenylindole (DAPI; Hoffmann-La Roche) for 40 min. Finally, cells were washed with PBS for 20 min and mono-distilled H_2_O for 10 s before mounting with Mowiol (Carl Roth) on glass slides (R. Langenbrinck). The prepared samples were dried over night at 4 °C and stored at the same temperature until analysis within 1 week. Mouse blastocysts were fixed and incubated with antibodies as described previously [11]. Mouse polyclonal pan cytokeratin antibody cocktail (PAN-CK) was from Thermo Fisher Scientific. Mouse monoclonal antibodies against Pkp1 (PP1-2D6), Pkp2 (PP2-86) and Pkp3 (PP3-270) as well as guinea pig polyclonal antibodies against DspI (DP-1) were from Progen Biotechnik. Rabbit polyclonal antibodies against Dsg2 were described previously [36]. Alexa Fluor 488- and 647-conjugated secondary goat antibodies were from Invitrogen. Cells transfected with fluorophore-tagged proteins that were not used for immunostainings were fixed with 4% (w/v) paraformaldehyde (Merck) in PBS (pH 7.2–7.4; adjusted with NaOH at max. 60 °C) for 15 min at room temperature. Cells were then washed for 5 min in PBS followed by a wash step in mono-distilled H_2_O for 10 s and were mounted in Mowiol.

### Microscopy

Microscopical recordings were performed with a laser scanning confocal microscope (LSM 710; Carl Zeiss) using Zen black 2.1 SP3 software (Carl Zeiss). The microscope was equipped with an Airyscan detector, oil immersion objective (63×/1.40-N.A. DIC M27) and a focus-shift correction system (DefiniteFocus; all from Carl Zeiss). For live-cell imaging, the microscope was pre-warmed to 37 °C and a 5% CO_2_ humidified atmosphere was used. Living HaCaT cells were imaged in glass-bottom dishes (12 mm glass-diameter, thickness 1.5#, MatTek) in 25 mM HEPES-buffered DMEM without phenol red (Life Technologies) supplemented with 2% fetal bovine serum. Living embryos were imaged in glass bottom dishes in M2 medium (Sigma-Aldrich) overlaid with mineral oil. Fluorescent reporter protein dynamics were recorded in HaCaT cells with the Airyscan detector in “resolution vs. sensitivity” mode. Immunostainings and reporter fluorescence in fixed cells were recorded in “super resolution” mode. The acquired signals were stored in a 16-bit data format before deconvolution with the help of Zen black software using automatic settings.

For detection of mCerulean in living cells an argon-ion laser (module LGK 7872 ML8) was used at 458 nm and 2.0–25.0% power together with a BP 420–445 + BP 465–505 filter. For detection of EYFP in living cells the argon-ion laser was used at 514 nm and 0.4–2.0% power together with a BP 495–550 + LP 570 filter. For detection of mApple and mCherry in living cells a 543 nm HeNe-laser (module LGK 7786 P) was used at 6.0–10% power without additional filters. In general, the detector gain was set to 850–925 and the samples were scanned at 1.59–4.70 µs pixel dwell time. The *z*-resolution was set to 0.25 or 0.34 µm and the pinhole to 175–206 µm. Note that mCerulean and mApple/mCherry were scanned together in one scan by alternated illumination, which greatly increased co-localization precision and speed.

In fixed cells DAPI fluorescence was recorded with a 405 nm diode laser, Alexa Fluor 488 with the argon-ion laser at 488 nm and Alexa Fluor 647 with a 633 nm HeNe-laser. Gain and laser intensities were optimized for best image quality or were identical for quantitative analysis.

For detection of EYFP in living embryos, the argon-ion laser was used at 488 nm and 0.3–2% power without additional filters. Detector gain was set to 800 and the samples were scanned at 1.58–6.46 µs pixel dwell time. The *z*-resolution was set to 0.173 µm, the pinhole to 117 µm and the Airyscan detector to “super resolution” mode.

In FRAP experiments, cells were recorded in three dimensions before and after bleaching at 3-min time intervals with the Airyscan detector set to standard confocal mode (*x* = 33.67 µm, *y* = 33.67 µm, *z* = 0.748 µm, 512 × 512-pixel resolution, pixel dwell time 1.58 or 2.54 µs). For recording the argon-ion laser was used at 488 nm at 0.1–0.3% power. Bleaching was triggered automatically 3 min after starting the first *z*-stack within an area of 5.28 × 5.28 µm or alternatively within stripes of 0.33 µm thickness of variable length. For bleaching the laser was increased to 100% power and the target area was scanned 40 times. Note that the bleaching took 10–35 s for the stripes depending on their length and 10 s for the square area, resulting in a small inaccuracy between time points 0 and 3 min after start of bleaching.

### Image analysis and statistical analysis

Microscopy images were processed and analyzed in Fiji distribution of ImageJ software package [37, 38]. The subsequent translocation of fluorescence from the unbleached cell part to the bleached region was measured as described in Ref. [32]. Statistics were calculated with Prism 5.01 (GraphPad). Curve fitting and regression were performed in SigmaPlot 12.0 (Systat). Figures were prepared with Adobe Photoshop and Illustrator CS 6 (Adobe). Movies were encoded in h.264 video format using Handbrake 1.1.2 software (https://handbrake.fr/).

## Results

### Keratins accumulate at desmosomal contact sites prior to keratin filament formation in early murine embryos

**Fig. 1:**
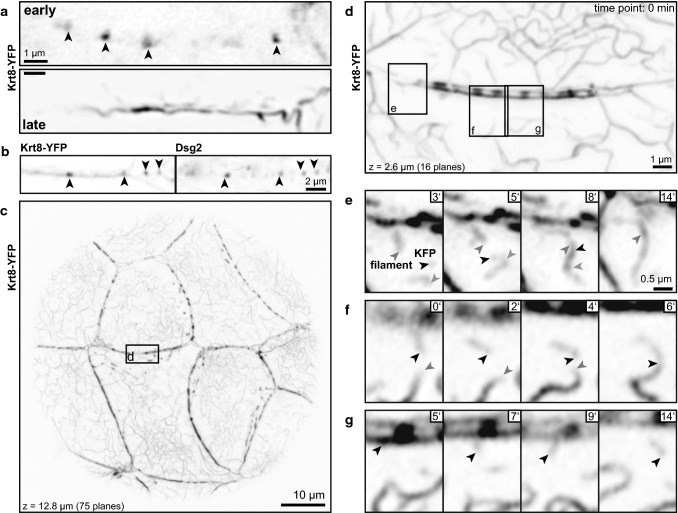
Keratins accumulate at desmosomes, interconnect desmosomes and polymerize into filaments next to desmosomes in murine blastocysts. The fluorescence micrographs (inverse presentation) were recorded in blastocysts of Krt8-YFP knock-in mice producing EYFP-tagged keratin 8 from the endogenous gene locus. They show details of keratin distribution and dynamics in the trophectoderm of living (**a**, **c**–**g**) and formaldehyde-fixed blastocysts (**b**). **a** The images show Krt8-YFP fluorescence at the cell border between adjacent trophectoderm cells appearing as punctate accumulations in an early blastocyst and in a pearls-on-a-string pattern consisting of elongated puncta that are connected by subcortical filaments in a late blastocyst. **b** Immunofluorescence microscopy of a cell border revealing localization of keratin puncta next to the desmosomal marker Dsg2 (arrowheads). **c** Shows an overview of the Krt8-YFP fluorescence in the lower part of a late blastocyst. The boxed area was imaged at higher magnification in Movie 1 and (**d**–**g**) to investigate KF formation. **d**–**g** The pictures are taken from Movie 1 showing keratin dynamics in the trophectoderm of a late blastocyst. **d** Depicts the typical pearls-on-a-string pattern of cortical KFs together with the newly formed cytoplasmic KF network and small keratin particles. **e**–**g** Highlight aspects of the dynamic behavior of keratin particles that are generated in the vicinity of the prominent keratin puncta at the plasma membrane. The newly formed keratin particles elongate and fuse with each other. Ends of single keratin particles are demarcated by arrowheads and tracked at later time points. Images **a**, **b**, **f**, **g** are single confocal planes, the images in **c**–**e** are maximum intensity projections. The background in **b** was filtered with a Gauss filter (strength set to 2.0 in Fiji). The gamma in **c** was adjusted to 0.4 and in **d** to 0.6 in Photoshop to increase signal intensity of thinner filaments

Using previously established knock-in keratin 8-EYFP (Krt8-YFP) reporter mice allows in vivo imaging of keratin network morphogenesis. In pre-implantation embryos, Krt8-YFP accumulates first in distinct dots at cell borders during the morula stage (embryonic day 3; [11]). The top image in Fig. 1a presents the dotted Krt8-YFP fluorescence in the trophectoderm of an early blastocyst. At later time points, subcortical KFs with local KF accumulations are detectable with very few cytoplasmic KFs (Fig. 1a, bottom). Immunostaining showed that the dotted keratin accumulations at the plasma membrane localize immediately next to the desmosomal protein Dsg2 (Fig. 1b). In conjunction with our previous observation, which demonstrated that these dots are also positive for desmoplakin (Dsp; [11]), we conclude that they correspond to desmosomal adhesion sites.

High-resolution time-lapse fluorescence recordings of late Krt8-YFP-blastocysts further revealed the appearance of small keratin particles emanating from the juxtamembraneous keratin–desmosome scaffold. To make sure that the appearance of particles was not caused by movement of filaments in and out of the focal plane, planes above and below were visually examined. These filamentous particles either remain attached to the plasma membrane or are released into the cytoplasm. They elongate and fuse into longer filaments, which eventually integrate into the cytoplasmic network (Fig. 1c–g; Movie 1). The keratin particles display hallmark features of the previously described KF precursors and squiggles [39, 40]. These highly dynamic filamentous rodlets originate from spherical particles that appear in close proximity to the plasma membrane [40].

Taken together, our findings suggest that desmosomes serve an important function in keratin network morphogenesis in vivo. To study details of this interaction, we decided to use a cell culture model to simultaneously monitor the dynamics of desmosomal protein organization and keratin network formation by double fluorescence microscopy.

### HaCaT keratinocytes establish a typical desmosome-anchored rim-and-spokes keratin system

Human immortalized HaCaT keratinocytes [31] were selected to examine keratin–desmosome interactions because they are well established as a model to study epidermal differentiation and have been used to interrogate keratin and desmosome dynamics (e.g., [6, 32, 41, 42]). Immunofluorescence microscopy showed that 3-day-old HaCaT monolayers consist of flattened cells with a nicely extended KF network presenting radial filaments that are anchored to DspI-positive desmosomes at cell–cell borders (left column of Fig. S1). After reaching confluence on day 5, cells start to grow on top of each other forming an incomplete multilayer by day 7 (middle and right columns of Fig. S1). Desmosomes are hyperadhesive at this time [42]. Between days 5 and 7 an increasing number of well-aligned desmosomal arrays could be visualized en face in projection views. These desmosomal arrays are connected by KFs, which are part of a subcortical network. We have recently proposed that this subcortical KF network cooperates with radial KFs to endow epithelial cells with unique biomechanical properties [43]. Optical resolution of the keratin–desmosome scaffold, however, turned increasingly difficult with prolonged culture time because of superposition of the fluorescence patterns in overlapping cells.

**Fig. 2:**
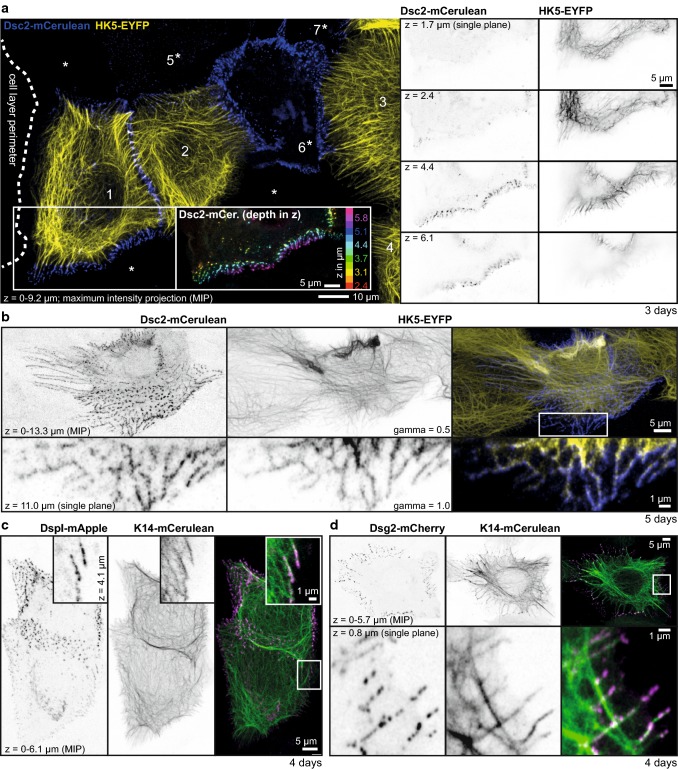
Radial and subcortical keratin filaments are firmly anchored to desmosomes in living HaCaT keratinocytes. The fluorescence micrographs depict different aspects of keratin–desmosome organization in living HaCaT keratinocytes producing fluorescent reporters. **a** Depicts at left the projection view of the fluorescence recorded in the outer part of an expanding cell colony (border demarcated by dotted line) consisting of wild-type HaCaT cells (denoted by asterisk) and HaCaT B10 cells producing fluorescent keratin 5 chimera HK5-EYFP (cells 1–4) that had been mixed in a 1:10 ratio at the time of seeding. The co-cultures were transfected on day 2 with an expression construct encoding fluorescent Dsc2 reporter Dsc2-mCerulean, which was detected on day 3 in cells 1, 2, 5, 6 and 7. The inset shows a color-coded view of the Dsc2-mCerulean fluorescence along the *z*-axis of the boxed area revealing predominant localization in upper focal planes. The single focal plane recordings at right (inverse presentation) further highlight the restricted distribution of desmosomes and the layer-specific KF network organization. **b** The fluorescence in a confluent HaCaT-derived cell culture that had been prepared in the same way as in **a** but was imaged 2 days later. By this time, cells had partially grown on top of each other. The projection views at the top depict a HK5-EYFP- and Dsc2-mCerulean-positive cell in the center presenting an extensive subcortical interdesmosomal keratin network (see also enlargement of a single focal plane of the boxed area at the bottom) and radial filaments that can be best appreciated in corresponding Movies 2 and 3 presenting rotating 3D views and animated *z*-stack series of a survey and detail. Corresponding Movie 4 depicts the dynamic changes of the fluorescence pattern over time. **c**, **d** Co-localization of DspI-mApple and Dsg2-mCherry with keratin 14-mCerulean in confluent HaCaT-derived cultures 4 days after seeding and 2 days after transfection with suitable expression constructs reveals distribution patterns similar to those seen in **a**, **b**. Enlarged views (single confocal planes) of the boxed areas are shown in the upper right corners of **c** and at the bottom of **d**, respectively. Movie 5 highlights dynamic details of the cells shown in **c**. Gamma was adjusted to 0.5 in Zen software in the middle top image of **b** to increase intensity of thinner filaments

To study keratin–desmosome interaction in living HaCaT cells, we employed subclone HaCaT B10 producing fluorescent keratin 5 (HK5-EYFP; [32]) and transfected them with expression constructs encoding fluorescent desmosomal protein reporters. To enable improved visualization of desmosome-anchored KFs within confluent HaCaT monolayers, we added wild-type HaCaT cells. In this way, we were able to detect and monitor single KFs and KF bundles together with their attached desmosomes (Fig. 2; Movies 2–5). As expected, most, if not all, of the Dsg2, Dsc2 and DspI reporter-positive cell contacts were linked to KFs. 3D reconstructions further revealed that desmosomes were primarily localized in the upper parts of cell monolayers (color-coded desmosome positioning and *z*-stack in Fig. 2a; animated *z*-stacks in Movies 2 and 3) in agreement with the results obtained by immunofluorescence microscopy (Fig. S1). Radial desmosomal KFs (spokes) were most prominent at day 3 (Fig. 2a). In addition to the radial KFs subplasmalemmal keratins connecting desmosomes to each other interconnected cortical networks (rim) were readily detectable on days 4–5 (Fig. 2b–d; Movies 2 and 3). The animated focal image series in Movie 3 illustrates that the rows of desmosomes are not cytoplasmic but localized at cell–cell borders since KFs from both adjacent cells insert into the same labeled desmosomes. Time-lapse fluorescence imaging further revealed that radial and subplasmalemmal keratins are permanently associated with desmosomes moving together as a structural unit (Movies 4 and 5). Formation of single desmosomes and events of associating/dissociating KFs could not be identified in these recordings.

### Nascent desmosomes serve as nucleation sites for elongating keratin filaments

To identify situations and loci, which would facilitate the study of sequential keratin–desmosome formation and maturation, we examined the distribution of the different Pkp isoforms in expanding HaCaT colonies. It has been shown that Pkp3 is incorporated into newly forming desmosomes [6, 25] together with or followed by Pkp2 [26, 44], whereas desmosomal Pkp1 heralds desmosome stabilization and maturation [6]. Using isotype-specific antibodies, we found that Pkp3 localized exclusively to DspI-positive desmosomes in the outermost periphery of HaCaT colonies 3 days after seeding (Fig. S2). Pkp3 fluorescence appeared to be substituted by Pkp2 fluorescence at anti-DspI-positive sites that were located closer to the center of the colonies including the region of zippering known to be involved in tight and stable cell–cell contact formation through membrane sealing (Fig. S2; [17]). Quantitative measurements showed that the outermost Pkp3/DspI-positive dots were significantly closer to the colony perimeter than the outermost Pkp2/DspI-positive dots (median of 3.7 ± 0.2 µm versus 6.3 ± 0.4 µm [SEM; *n* = 120 (adjacent cell borders); *p* < 0.0001, Wilcoxon matched pairs test two-tailed]). Furthermore, Pkp1 was virtually absent in peripheral cells but could be detected in suprabasal cells of confluent cell cultures (Fig. S2). We therefore conclude that new desmosomes are formed in the most peripheral domains of expanding HaCaT colonies at cell–cell borders.

**Fig. 3:**
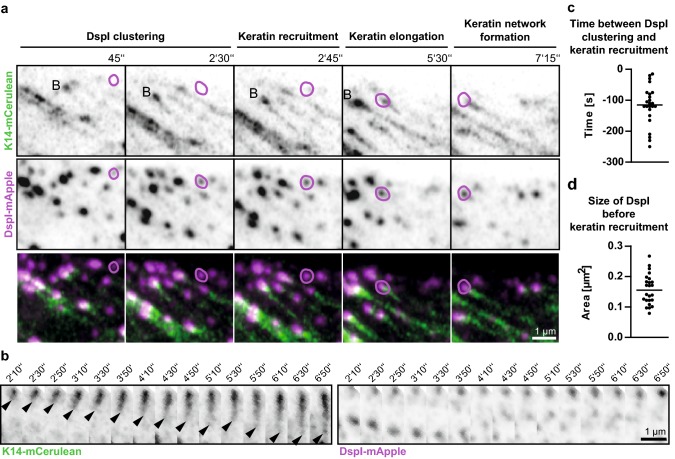
Keratin filaments nucleate at and grow from newly formed desmoplakin I-positive sites. **a**, **b** The images show K14-mCerulean fluorescence together with DspI-mApple fluorescence in cDNA-transfected HaCaT cells. They were recorded at the periphery of expanding HaCaT colonies. The entire time-lapse series is provided in Movie 6. The selected images exemplify that DspI-mApple accumulates first at cell–cell contact sites followed by keratin recruitment. The desmosomal keratin accumulations are then used as nuclei for elongating KFs, which subsequently fuse and connect to the KF network. The process of keratin nucleation and KF elongation is depicted in more detail in **b**. **c** The scatter plot shows that DspI clusters appear 115 ± 13 s (SEM) before nearby keratin enrichment becomes visible (*n* = 24 events). **d** The scatter plot reveals that DspI clusters have a median size of 0.16 ± 0.01 µm^2^ (SEM) at the time of keratin recruitment (*n* = 24 events)

**Fig. 4:**
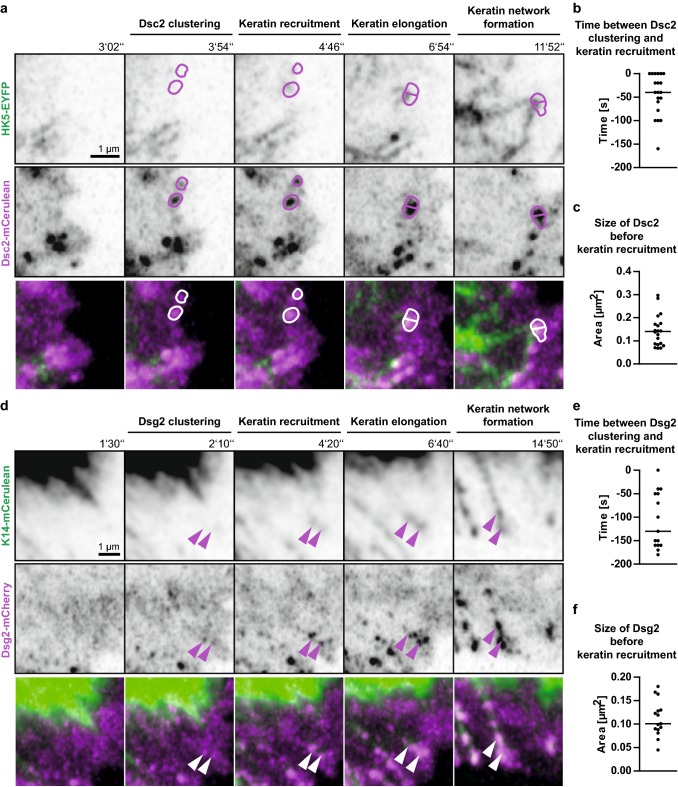
Keratin filaments nucleate at and grow from newly formed desmocollin 2- and desmoglein 2-positive sites. **a** The images highlight stages of Dsc2-mCerulean clustering, HK5-EYFP recruitment, KF elongation from desmosomal nucleation sites and KF network formation in living HaCaT cells mixed with HaCaT B10 cells at day 3 after plating (see also corresponding Movie 7). **b** The scatter plot shows that Dsc2 clusters appear 40 ± 10 s (SEM) before nearby keratin enrichment becomes visible (*n* = 20 events). **c** The scatter plot reveals that Dsc2 clusters have a median size of 0.14 ± 0.02 µm^2^ (SEM) at the time of keratin recruitment (*n* = 20 events). **d** The selected fluorescence micrographs depict stages of Dsg2-mCherry clustering, K14-mCerulean recruitment, KF elongation from desmosomal nucleation sites and KF network formation in HaCaT cells mixed with HaCaT B10 cells at day 3 after plating (see also corresponding Movie 8). **e** The scatter plot shows that Dsg2 clusters appear 130 ± 15 s (SEM) before nearby keratin enrichment becomes visible (*n* = 15 events). **f** The scatter plot reveals that Dsg2 clusters have a median size of 0.10 ± 0.01 µm^2^ (SEM) at the time of keratin recruitment (*n* = 15 events)

The next set of experiments focused on the dynamic properties of fluorophore-tagged desmosomal proteins in relation to fluorophore-tagged keratins in the periphery of expanding HaCaT colonies. A typical example is shown in Fig. 3 and corresponding Movie 6. The selected region depicts newly forming DspI clusters, one of which is labelled in the middle panel of Fig. 3a. Within 2 min after the appearance of the DspI cluster partially juxtaposed keratin fluorescence becomes detectable (2 min 45 s). Subsequently, keratin fluorescence extended away from the DspI-positive cluster forming a new KF (5 min 30 s) that connected to the cytoplasmic keratin network (7 min 15 s). Figure 3b highlights another example of desmosome-dependent KF growth. Keratin accumulation and filament elongation could also be detected next to nascent Dsc2- and Dsg2-positive clusters at peripheral adjoining cell borders of expanding HaCaT colonies (Fig. 4a, d; Movies 7 and 8). We visually examined in each instance the focal planes below and above to ascertain that desmosomal protein and keratin clusters were formed only in the recorded plane and were not caused by movement of preformed particles.

Analyses of the sequence of events showed that the time between desmosomal protein accumulation and keratin recruitment differed significantly between Dsc2 and DspI as well as between Dsc2 and Dsg2 (1-way ANOVA, Kruskal–Wallis test with Dunn’s post test, 95% confidence intervals). The time interval was longest for Dsg2 [130 ± 15 s (SEM); *n* = 15 (early desmosomes); Fig. 4e], shortest for Dsc2 (40 ± 10 s; *n* = 20; Fig. 4b) and intermediate for DspI (115 ± 13 s; *n* = 24; Fig. 3c). We therefore conclude that Dsg2 clusters first, followed by DspI recruitment and subsequent co-clustering with Dsc2. Since we did not directly compare the clustering events, we cannot exclude that alternative sequences of events occur occasionally. Be it as it may, KF recruitment always occurred after desmosomal proteins had clustered and we never found desmosomal cluster formation on pre-existing KFs in the cell periphery. We finally analyzed desmosomal protein cluster size at the time of keratin recruitment. Median Dsg2 cluster size was 0.10 ± 0.01 µm^2^ (SEM; Fig. 4f), Dsc2 cluster size was 0.14 ± 0.02 µm^2^ (Fig. 4c) and DspI cluster size was 0.16 ± 0.01 µm^2^ (Fig. 3d). These dimensions classify the desmosomes as very small (cf. [9]) as would be expected of nascent desmosomes.

Taken together, we conclude that desmosome formation and KF formation are spatially linked and occur sequentially.

### Desmosome-attached keratin filaments bundle coinciding with reduced turnover of keratins and desmoplakin

**Fig. 5:**
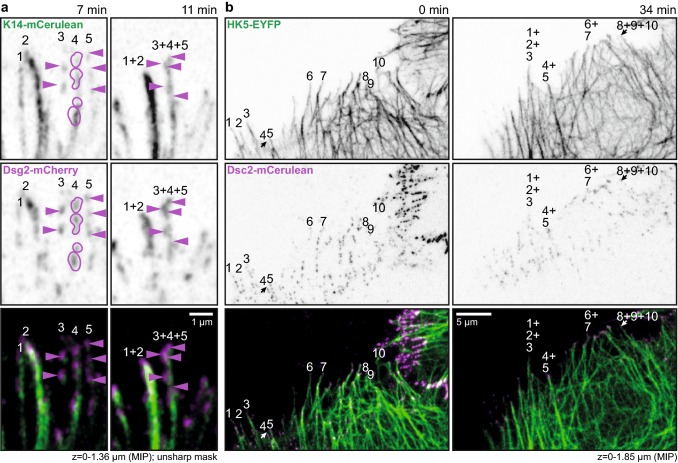
Desmosome-attached keratin filaments merge into bundles. The fluorescence images were recorded 3 days after seeding and were taken from time-lapse recordings provided as Movies 9 and 10. **a** HaCaT cells had been co-transfected with expression constructs encoding keratin 14-mCerulean and Dsg2-mCherry. Arrays of desmosomal and interdesmosomal keratins are numbered, desmosomal Dsg2-positive clusters are marked by arrowheads. Note the fusion of KFs resulting in intercalation of associated Dsg2 clusters. **b** HaCaT B10 cells producing HK5-EYFP were mixed with wild-type HaCaT cells and transfected with an expression construct encoding Dsc2-mCerulean. Some interdesmosomal and radial KFs, which fuse during the time of recording, are numbered. Note that these events lead to desmosomal fusion and intercalation

Desmosomal KF morphogenesis continues after linkage. Two different processes can be distinguished afterwards (Fig. 5a, b; Movies 9 and 10): (i) interdesmosomal cortical KFs merge into thicker filament bundles. As a result desmosomes intercalate and become more closely spaced. (ii) Radial desmosome-anchored KFs merge into thick KF bundles. At the same time the associated desmosomes fuse with each other resulting in larger desmosomes. Both systems together are the basis of the thick, desmosome-anchored keratin bundles seen in the elongated cytoplasmic bridges connecting suprabasal cells of partially multilayered HaCaT cultures at day 6 after seeding (e.g., Fig. S3). This arrangement is reminiscent of the arrangement found in the spinous layer of the epidermis.

**Fig. 6:**
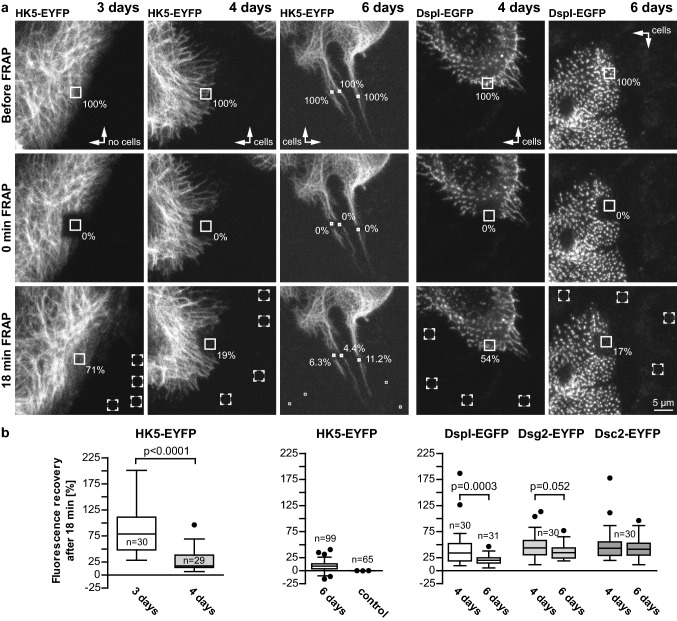
The turnover of desmosome-anchored keratin filaments and desmosomal desmoplakin decreases over time. FRAP experiments were performed to determine the turnover of KFs and desmosomal proteins in HaCaT cells at different times after seeding. **a** Typical examples of the fluorescence before bleaching (pre-bleach), immediately after bleaching (0 min post-bleach) and at the end-point (18 min post-bleach) are shown from top to bottom. Note that the entire bleached area was larger than the region of interest (solid box) that was used for calculating the fluorescence recovery to avoid effects of cell movement during the recording period. The first column shows FRAP of the peripheral cytoplasm of a cell at the perimeter of an expanding HaCaT colony 3 days after seeding, the second column shows FRAP of desmosome-anchored KFs in a confluent HaCaT monolayer 4 days after seeding, and the third column shows FRAP of desmosome-anchored KFs in cytoplasmic bridges between HaCaT cells 6 days after seeding that had partially grown on top of each other. The fourth and fifth columns depict the results of DspI-EGFP FRAP 4 and 6 days after seeding. The results for similar FRAP experiments of Dsc2-EYFP and Dsg2-EYFP are included in the whisker box plots in **b**. **b** Quantification of multiple separate experiments as box plots, whiskers are Tukey. Statistical analysis was performed with a two tailed Wilcoxon matched pairs test. *n* number of cells except for HK5-EYFP FRAP measurements after 6 days, where *n* refers to the number of filament bundles examined

To assess the consequences of this maturation process on protein turnover, fluorescence recovery after photobleaching (FRAP) experiments were performed (Fig. 6a). The highest keratin turnover was observed in the peripheral cytoplasm of cells located at the periphery of expanding HaCaT colonies at day 3 [78.8 ± 8.5% (SEM) after 18 min; Fig. 6b]. The median keratin turnover was drastically decreased in the peripheral cytoplasm of cells that were connected through desmosomes 4 days after seeding (17.4 ± 4.1% after 18 min; *p* < 0.0001). The lowest turnover was observed within the cytoplasmic keratin-rich bridges 6 days after seeding (8.9 ± 0.9% after 18 min). In the next set of experiments, we investigated how desmosomal protein turnover was affected by increasing time after seeding (Fig. 6a, b). Fluorescence recovery of DspI-EGFP was significantly reduced from 34.2 ± 6.7% (SEM) at day 4 to 19.9 ± 1.7% at day 6 (*p* = 0.0003) after 18 min. The turnover of fluorescent Dsg2 and Dsc2 reporters, however, was not significantly reduced after 18 min (43.7 ± 4.4% versus 34.5 ± 2.6% for Dsg2-eYFP; 43.2 ± 5.9% versus 41.2 ± 3.7% for Dsc2-eYFP). Additionally, comparison between the kinetics of these desmosomal-proteins at 3-min time intervals show highest turnover for Dsc2, closely followed by Dsg2, and slower turnover for DspI which is especially noticeable at day 6 (Fig. S4).

We therefore, conclude that desmosomal anchorage together with subsequent desmosome fusion and KF anchorage results in stabilization of keratin–desmosome connectivity through reduced protein turnover without affecting turnover dynamics of the desmosomal transmembrane core components.

## Discussion

By examining keratin dynamics in conjunction with spontaneously occurring desmosome formation in expanding HaCaT keratinocyte colonies, we were able to detect keratin nucleation at nascent desmosomes, formation of an interdesmosomal network and bundling of desmosome-anchored KF. The importance of desmosomes for keratin network organization was confirmed by observations in vital murine blastocysts. These findings provide long-sought links within the functionally and structurally tightly coupled keratin–desmosome scaffold that is a fundamental prerequisite of epithelial resilience (recent reviews in Refs. [43, 45, 46]).

**Fig. 7:**
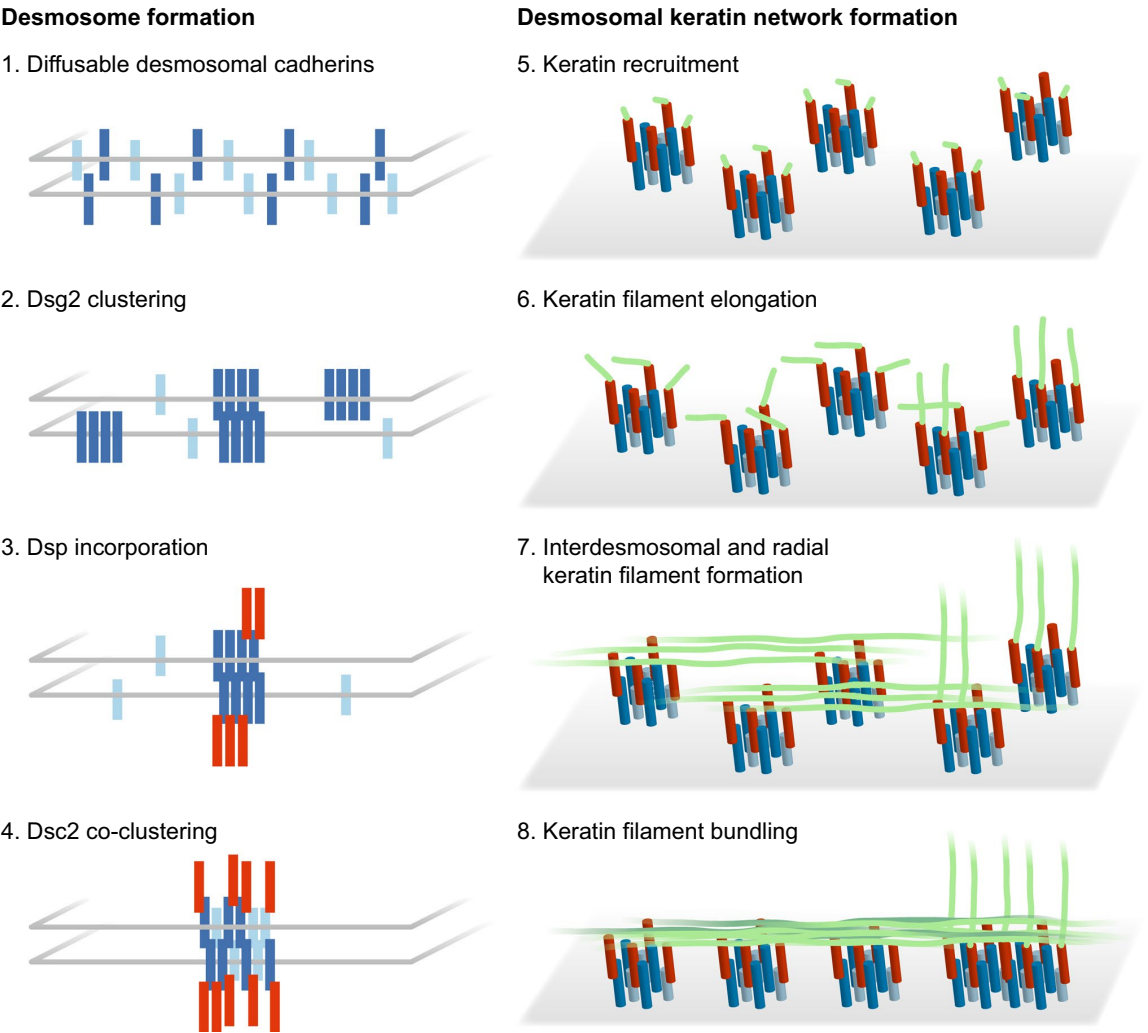
The scheme summarizes major steps of keratin–desmosome scaffold formation. The left part shows the order of desmosomal protein clustering as determined in the current study. The right part depicts subsequent desmosomal keratin filament nucleation, elongation and bundling leading to the formation of a desmosome-anchored rim-and-spokes system. Further details in the text

Although it is known that keratin networks and desmosomes form on their own [15, 17, 18, 47, 48], it has remained a conundrum, precisely when and where keratin filaments and desmosomes attach to each other to form the mechanically resilient transcellular network in epithelial tissues. It has been suggested that polymerized keratins and KF bundles associate with nascent desmosomes or desmosomal precursors [26, 44, 49, 50]. We now present in vitro and in vivo evidence for an alternative mechanism involving nucleation and subsequent elongation of KFs at desmosomes. This mechanism appears to be most relevant in situations of de novo KF–desmosome formation as is the case during tissue development and in epithelial expansion occurring during colony growth in vitro. It allows topologically defined keratin network growth with subcellular precision. Dissecting the sequence of events from desmosomal protein clustering to KF elongation presented a formidable challenge because of the restricted occurrence of this process in defined subcellular domains and the speed of individual assembly steps taking just a few seconds in a rapidly changing 3D environment. In the past it was therefore difficult for us and others to capture keratin–desmosome scaffold morphogenesis at sufficient resolution and with the necessary reproducibility in classical experimental setups such as calcium-switching, obstacle removal and scratch-wounding (e.g., [13, 26]). Examining the outermost adjacent plasma membrane regions of peripheral cells in expanding cell colonies proofed to be crucial in the current study to reproducibly detect successive stages of desmosome and keratin filament assembly. Mixing labeled with non-labeled fluorescent reporter cells further helped to improve the resolution and mapping precision of this process in individual cells. In this way, we were able to define a sequence of events starting with Dsg2 clustering followed by DspI recruitment, Dsc2 incorporation, keratin accumulation and KF elongation (summary scheme in Fig. 7). These observations extend and further refine previous observations by others [19, 51] showing that 5 min after E-cadherin clustering Dsp accumulates together with Dsg2 at new cell contacts during what the authors referred to as phase I of desmosome formation. In addition, previous reports have demonstrated that Dsp recruitment to cell contacts involves Pkp2 and Pkp3 [6, 26], which is in agreement with our observation of Pkp3 at the most peripheral part of HaCaT colonies and subsequent replacement by Pkp2. The recruitment of Pkp1 is obviously a much later phenomenon that is related to cytoskeletal maturation and linked to desmosomal hyperadhesion [6, 52].

Probably the most enigmatic finding of our present study was desmosomal nucleation of KFs raising questions about the mechanisms facilitating tethering of non-filamentous keratins to nascent desmosomes. Since we see keratin accumulation only after clustering of desmosomal proteins including the keratin binding proteins Dsp and Pkp [1, 53–56], either the assembly of desmosomal proteins themselves serves as a template for keratin nucleation or other additional factors are activated/recruited to elicit keratin accumulation. 14-3-3 proteins may be such factors. They have been shown to be involved in mechanosensitive keratin recruitment toward cell–cell contacts in migrating mesendoderm of the *Xenopus laevis* gastrula [57]. Given the evolutionary diversity and cell type-specificity of desmosome and KF composition these or other factors may drive this process in the different environments. The relevance of desmosomal KF nucleation during steady state is presently unknown. FRAP experiments in embryos, however, revealed that desmosomal puncta appear prior to interdesmosomal and cytoplasmic filaments [11].

Elongation of desmosome-anchored KFs may occur either by addition of subunits at the desmosomal plaque or addition of subunits to the extending filament tips. Given the lack of intrinsic KF polarity both mechanisms are likely to take place as has been observed for growing keratin particles in single cells [40]. The comparatively high turnover of desmosomal keratins noted in developing murine embryos [11] and the unstructured, i.e., non-filamentous appearance of desmosomal keratins (this study and [11]) both suggest that soluble keratins are recruited to desmosomal nucleation sites. It is assumed that the soluble keratins are primarily composed of tetramers [58]. Whether and how these subunits assemble into unit length filaments and subsequently form elongated filaments remains to be elucidated [59].

Our observations further highlight the importance of an hitherto poorly characterized interdesmosomal keratin network. While punctate accumulation of keratins was first observed both in the developing blastocyst and in expanding epithelial colonies, it was followed in both instances by establishment of an interdesmosomal network prior to cytoplasmic network formation. The advent of improved 3D resolution has placed the focus on this understudied system only recently defining it as a biomechanically relevant cytoskeletal component with potential function in mechanosensing [43]. With the optimized recording setup, most of the presumptive cytoplasmic desmosomal dots were identified in the current study as plasma membrane-localized desmosomes that are connected by KFs. The en face views of this interdesmosomal KF network in our images were due to the fact that HaCaT keratinocyte monolayers do not consist of cylindrical cells as is the case, for example, for MDCK monolayers but grow partially on top of each other. Thus, several of the previous observations may require re-interpretation of cytoplasmic versus plasma membrane localization of desmosomal components. Our current double localization of keratins and desmosomal components now explains the previously observed coordinated motility of aligned desmosomes for over a day [13] and lack thereof in keratin-free keratinocytes [15].

Our focus on membrane-associated keratins further revealed merging of interdesmosomal KFs together with their associated desmosomes forming an extensive pearls-on-a-string pattern. In addition, fusion of radial desmosome-attached KFs was also observed. We suggest that these processes are steered by local strains and stresses to adapt the keratin–desmosome scaffold to specific mechanical loads and hence structural requirements. Upon completion of the adaptive response the scaffold becomes less dynamic as evidenced by the increased turnover times of its polypeptide components.

Taken together our work picks up on and revitalizes older work, which emphasized the importance of desmosomes as organizing centers for the keratin network. Mattey and Garrod [14] detected small desmosome-like adhesions with associated fine filaments 20 min after switch from low to standard calcium medium in Madin-Darby canine kidney MDCK cells by electron microscopy. They developed into a clearly recognizable desmosome-cortical keratin filament scaffold within the next 10 min. Bologna et al. [12] reported on keratin accumulation at newly formed desmosomes and subsequent KF extension in a rat mammary epithelial cell line. Technical limitations concerning antibody specificity, heterogeneity of staining patterns, limited temporal resolution and cell type-restricted occurrence prevented follow-up work. More recent work has shown that the cross-talk between keratins and desmosomes depends on the keratin isotype [60]. While we find similar desmosomal interactions for keratins 8/18 in murine blastocysts and keratins 5/14 in HaCaT keratinocytes, keratins 6/16/17 have been shown to behave differently [60]. These keratins are expressed during wound healing and decrease desmosome stability by PKCα recruitment. A full understanding of keratin–desmosome dynamics will have to take into consideration these and other factors such as p38 MAPK [61] which regulate keratin–desmosome attachment. A comprehensive understanding of the dynamic interaction of keratins not only with desmosomes, but also with hemidesmosomes will help to define the contribution of the junction-attached keratin network to the modulation of epithelial cell plasticity for maintaining epithelial homeostasis, supporting effective epidermal wound healing and enabling cancer metastasis.

## Electronic supplementary material

Below is the link to the electronic supplementary material.

Supplementary material 1 (MP4 2340 kb)

Supplementary material 2 (MP4 19471 kb)

Supplementary material 3 (MP4 2288 kb)

Supplementary material 4 (MP4 1780 kb)

Supplementary material 5 (MP4 2377 kb)

Supplementary material 6 (MP4 4481 kb)

Supplementary material 7 (MP4 6267 kb)

Supplementary material 8 (MP4 5456 kb)

Supplementary material 9 (MP4 2548 kb)

Supplementary material 10 (MP4 4847 kb)

Supplementary material 11 (PDF 4394 kb)

## Abbreviations

µm: Micrometer
3D: Three-dimensional space
cDNA: Complementary DNA
cf.: Compare
DAPI: 4′,6-Diamidino-2-phenylindole
DMEM: Dulbecco’s modified Eagle’s medium
DNA: Deoxyribonucleic acid
Dsc: Desmocollin
Dsg: Desmoglein
Dsp: Desmoplakin
e.g.: For example
EDTA: Ethylenediaminetetraacetic acid
EGFP: Enhanced green fluorescent protein
EYFP: Enhanced yellow fluorescent protein
Fig.: Figure
FRAP: Fluorescence recovery after photobleaching
HEPES: 4-(2-Hydroxyethyl)-1-piperazineethanesulfonic acid
i.e.: In other words
KF: Keratin intermediate filament
Krt: Keratin
MAPK: Mitogen-activated protein kinase
min: Minute
MIP: Maximum intensity projection
PBS: Phosphate-buffered saline
PCR: Polymerase chain reaction
Pg: Plakoglobin
Pkp: Plakophilin
s: Second
SEM: Standard error of the mean
