# Supplementary material for: The keratin–desmosome scaffold: pivotal role of desmosomes for keratin network morphogenesis
Source: Cell Mol Life Sci. 2019 Jun 26;77(3):543–58. doi: 10.1007/s00018-019-03198-y (PMC7010626; doi:10.1007/s00018-019-03198-y)
Supplement: Supplementary file 11 — Supplementary material 11 (PDF 4394 kb) [file 18_2019_3198_MOESM11_ESM.pdf]

Electronic supplementary material

The keratin-desmosome scaffold: Pivotal role of desmosomes for keratin network morphogenesis

Marcin Moch[a, b], Nicole Schwarz[a, b], Reinhard Windoffer[a], Rudolf E. Leube[a]

[a] Institute of Molecular and Cellular Anatomy, RWTH Aachen University, 52074 Aachen, Germany

[b] Authors contributed equally.

Corresponding author: Rudolf Leube

Institute of Molecular and Cellular Anatomy

RWTH Aachen University

Wendlingweg 2

52074 Aachen

Germany

Fax: +49 241 80 82508

Phone: +49 241 80 89107

Email: rleube@ukaachen.de

Table of Contents

Supplementary Figures..3
Movie Legends 6

Supplementary Figures

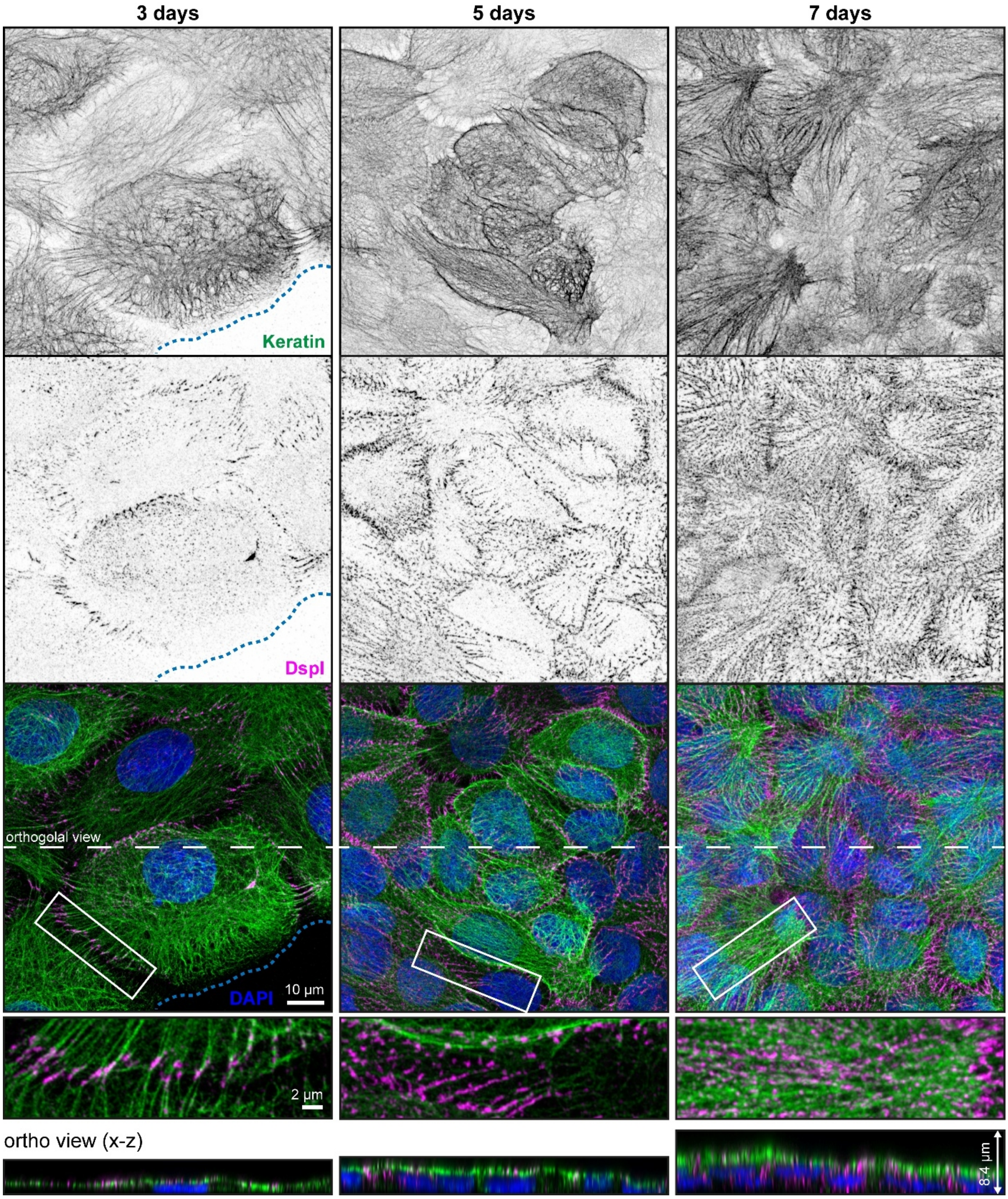

Fig. S1 The keratin-desmosome scaffold becomes increasingly complex over time in HaCaT keratinocytes. Double immunofluorescence microscopy was performed in HaCaT cells 3, 5 and 7 days after seeding detecting desmosomes by anti-DspI and keratins by anti-keratin staining. The images show maximum intensity projections of multiple planes at low magnification in the top 3 image rows and of the boxed areas at higher magnification in the fourth row. X-z orthogonal views along the broken lines marked in the third row are presented at the bottom. Note the predominance of radial desmosome-attached KFs at left and the increasing number of interdesmosomal KFs in the middle and at right, which are difficult to delineate at increasing cell density because cells start to grow on top of each other forming an incomplete double layer.

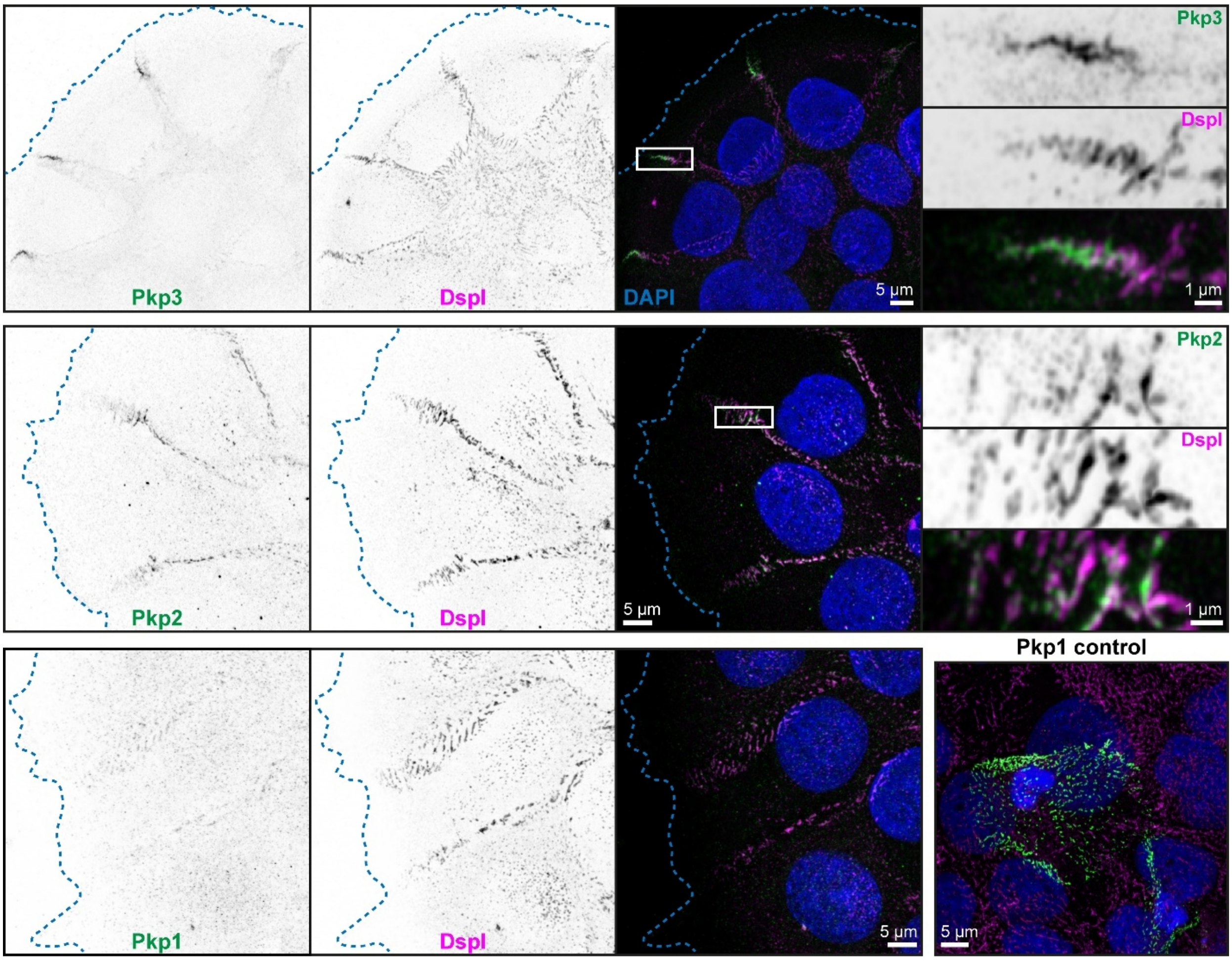

Fig. S2 Plakophilin isoforms are differentially distributed in desmosomes of HaCaT keratinocytes. The double immunofluorescence micrographs show the different Pkp isoforms in relation to DspI in expanding HaCaT colonies 3 days after seeding. Note that Pkp3 labels the most peripheral DspI-positive desmosomes and is replaced by Pkp2 further inward, while Pkp1 cannot be detected at this early time point after seeding. Pkp1 fluorescence is only noted at later time points in suprabasal HaCaT cells (bottom right; Pkp1 control at day 6).

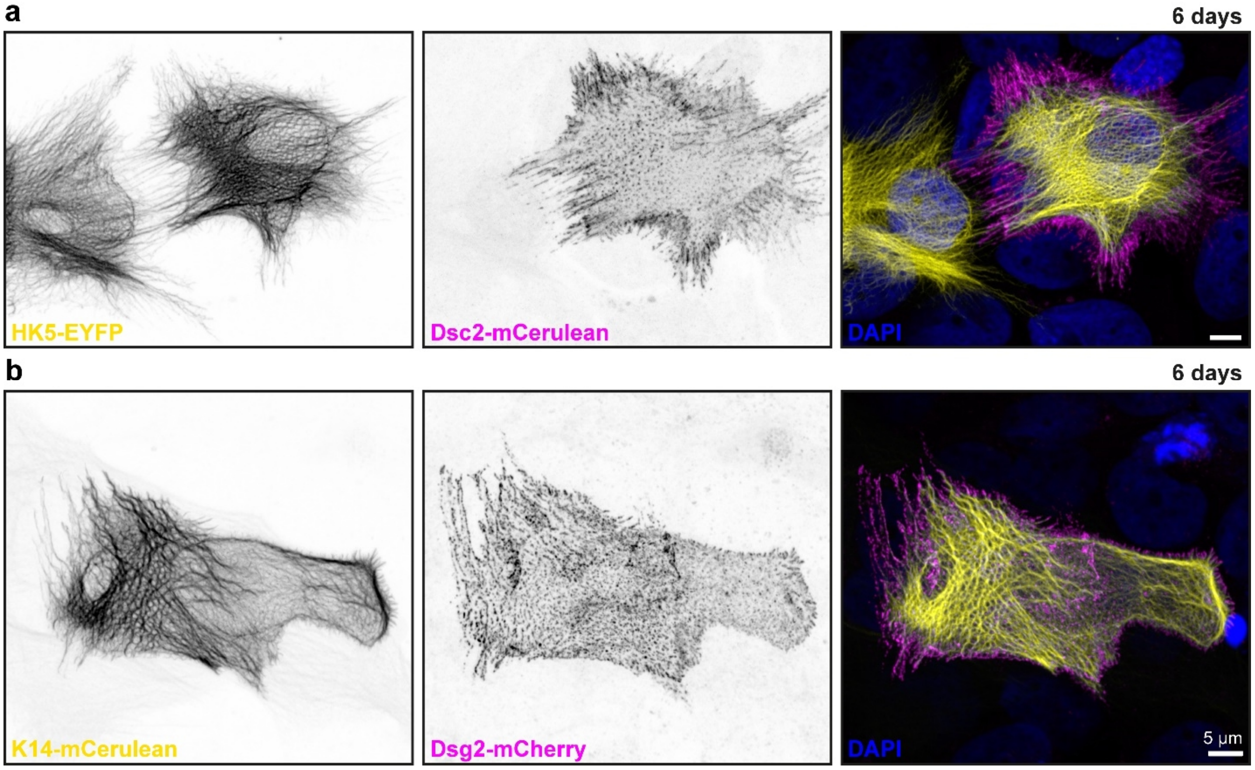

Fig. S3 Suprabasal cells are connected to cells below and to neighboring cells by a highly-ordered keratin-desmosome-scaffold. **a-b** The fluorescence images show HK5-EYFP/Dsc2-mCerulean and K14-mCerulean/Dsg2-mCherry distribution patterns in confluent, formaldehyde-fixed HaCaT keratinocytes at day 6 after seeding. Note that the transfected cells are completely surrounded by non-transfected cells (nuclei stained with DAPI). The images depict thick keratin filament bundles connecting highly-ordered rows of desmosomes.

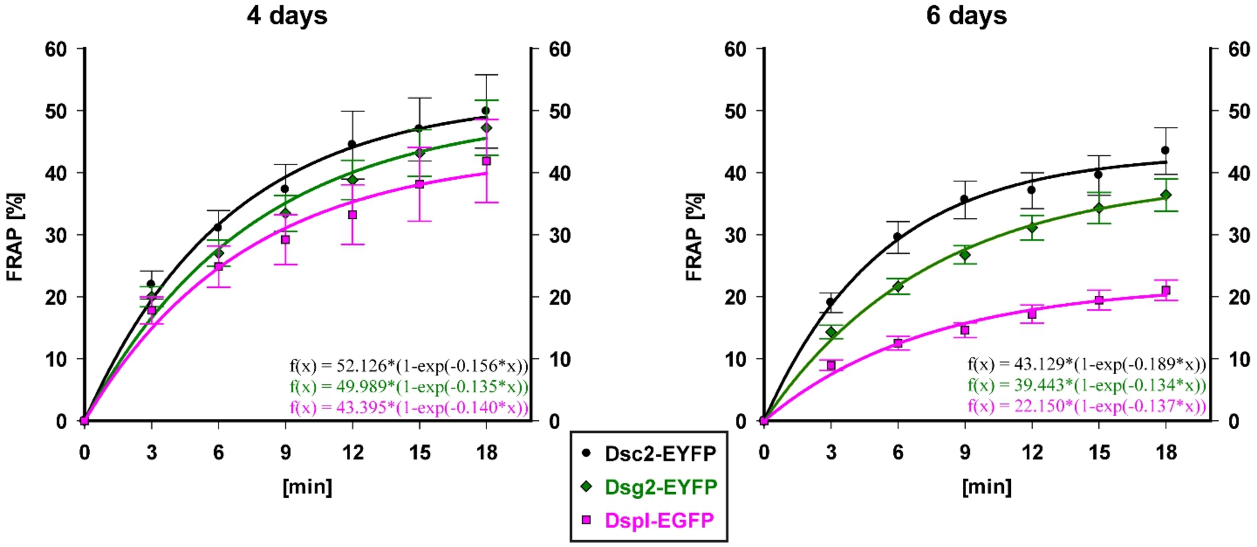

Fig. S4 Desmosomal-cadherins show higher turnover of the rapidly exchangeable pool than desmoplakin. The data correspond to those shown in Fig. 6b and show FRAP at 3 min intervals (n=30 cells except for FRAP measurements of Dspl-EGFP (n=31)). Curves were fitted to data in SigmaPlot (equation: exponential rise to maximum with 2 parameters). Data points: mean. Error bars: standard error.

Movie Legends

Movie 1 Keratin filaments nucleate close to the cortical interdesmosomal network in murine blastocysts. The overview at left shows an animated maximum intensity projection of the fluorescence recorded in the trophectoderm of a late murine Krt8-YFP knock-in blastocyst (see also corresponding Fig. 1c). The time-lapse fluorescence images at right were recorded at the cell border of two trophectoderm cells of the same blastocyst every 62.5 s (see also corresponding Fig. 1d). The top reveals the appearance of growing keratin particles from the interdesmosomal subcortical keratin system. These motile and highly flexible particles fuse and enlarge before merging with the cytoplasmic KF network (arrowheads delineate a few selected examples; contrast was enhanced per frame to compensate for bleaching). The lower plane shows the same region at enhanced contrast settings to unravel further details in the selected focal planes.

Movie 2 The 3D animation of a z-stack recording depicts the fluorescence of keratin and desmosome reporters in a confluent HaCaT cell culture. The images (maximum intensity projection) were obtained from mixed HaCaT cell cultures containing cells producing HK5-EYFP (clone B10) delineating KFs and Dsc2-mCerulean delineating desmosomes (for details see legend to corresponding Fig. 2b). The animation first shows rotating views of the keratin-desmosome fluorescence, then rotating views of the desmosome fluorescence by itself, and then top-to-bottom views of single focal planes of double keratin-desmosome fluorescence.

Movie 3 Scroll through confocal sections reveals organizational details of the keratin-desmosome scaffold in a confluent HaCaT cell culture. The images were obtained from HaCaT cells producing HK5-EYFP delineating KFs and Dsc2-mCerulean delineating desmosomes (for details see legend to corresponding Fig. 2b). Note the presence of desmosome-anchored interdesmosomal and radial KFs in different focal planes.

Movie 4 Time-lapse image series depicts the coordinated dynamics of HK5-YFP and Dsc2-mCerulean in a confluent HaCaT culture. The images (maximum intensity projections) were recorded every 55 s in HaCaT cells producing HK5-EYFP delineating KFs and Dsc2-mCerulean delineating desmosomes (for details see legend to corresponding Fig. 2b). Note the coordinated movement of desmosomes with attached subcortical and radial KFs.

Movie 5 The time-lapse image series depicts the coordinated dynamics of keratin 14-mCerulean and DspI-mApple in a confluent HaCaT cell culture. The images (maximum intensity projections; 45 s recording intervals) were obtained from HaCaT cells producing Keratin 14-mCerulean and DspI-mApple delineating KFs and desmosomes (for details see legend to corresponding Fig. 2c). Note the coordinated movement of desmosomes with attached subcortical and radial KFs.

Movie 6 Elongating keratin filaments nucleate at nascent desmoplakin clusters at the outermost cell border of an expanding HaCaT colony. The time-lapse series (maximum intensity projections; 15 s recording intervals) shows DspI-mApple and K14-mCerulean in two adjacent HaCaT cells. Examples of clustering DspI-mApple are delineated by magenta arrows, the ends of elongating KFs by green arrows. For further details see legend to corresponding Fig. 3.

Movie 7 Elongating keratin filaments nucleate at nascent desmocollin 2 clusters. The time-lapse series (maximum intensity projections; 26 s recording intervals) shows the fluorescence of Dsc2-mCerulean and HK5-EYFP at the expanding cell-cell border of a HaCaT cell next to a non-transfected HaCaT cell in the periphery of an expanding HaCaT colony. Examples of clustering Dsc2-mCerulean are delineated by magenta arrows, the ends of elongating KFs by green arrows. For further details see legend to corresponding Fig. 4a.

Movie 8 Elongating keratin filaments nucleate at nascent desmoglein 2 clusters. The time-lapse series (maximum intensity projections; 26 s recording intervals) shows the fluorescence of Dsg2-mCherry and Keratin 14-mCerulean at the expanding cell-cell border of a HaCaT cell next to a non-transfected HaCaT cell in the periphery of an expanding HaCaT colony. Examples of clustering Dsg2-mCherry are delineated by magenta arrows. For further details see legend to corresponding Fig. 4d.

Movie 9 Subcortical, interdesmosomal keratin filaments merge into bundles. The time-lapse series (maximum intensity projections; 60 s recording intervals) shows Keratin 14-mCerulean and Dsg2-mCherry fluorescence at the cell border in a HaCaT cell colony 3 days after seeding (see also corresponding Fig. 5a). Note the fusion of color-coded interdesmosomal KFs into KF bundles with tightly-spaced Dsg2-mCherry-positive desmosomes producing a pearls-on-a-string pattern.

Movie 10 Interdesmosomal and radial keratin filaments merge into bundles. The time-lapse series (maximum intensity projections; 26 s recording intervals) shows HK5-EYFP and Dsc2-mCerulean fluorescence at the cell border in a HaCaT cell colony 3 days after seeding (see also corresponding Fig. 5b). Note the fusion of color-coded interdesmosomal and radial KFs into KF bundles with tightly-spaced and enlarged Dsc2-mCerulean-positive desmosomes.
